# The Associations between Mental Well-Being and Adherence to Physical Activity Guidelines in Patients with Cardiovascular Disease: Results from the Scottish Health Survey

**DOI:** 10.3390/ijerph16193596

**Published:** 2019-09-26

**Authors:** Ahmad Salman, Maha Sellami, Abdulla Saeed AL-Mohannadi, Sungsoo Chun

**Affiliations:** 1 College of Health Sciences, QU Health, Qatar University, 2713 Doha, Qatar; 2 Department of Health Sciences, University of York, York YO10 5DD, UK; 3 Kuwait Public Policy Centre, General Secretariat of the Supreme Council for Planning and Development, 13001 Safat, Kuwait; 4 Sport Science Program (SSP), College of Arts and Sciences (CAS), Qatar University,2713 Doha, Qatar; 5 Research and Scientific Support Department; Aspetar, Orthopaedic and Sports Medicine Hospital, 29222 Doha, Qatar

**Keywords:** physical activity, mental well-being, health-related behavior

## Abstract

The association between physical activity (PA) and mental well-being in individuals with a cardiovascular disease (CVD) is poorly studied. The objective of this study was to assess the association between mental well-being and adherence to the recommended guidelines for PA in a Scottish adult population with CVD. The study used data from 3128 adults who had CVD conditions (1547 men and 1581 women; mean age 63.29 years) who participated in the Scottish Health Survey between 2014 and 2017. The Warwick–Edinburgh Mental Well-Being Scale (WEMWBS) was used as a surrogate measure of mental health. PA was classified as “met” or “unmet” on the basis of the recommended PA guidelines (150 min of moderate activity or 75 min of vigorous activity per week). The relationship between PA guidelines being met and the WEMWBS score was explored using hierarchical linear regression accounting for a set of health and sociodemographic characteristics. Of the participants, ~41.8% met the recommended PA levels. Among those with CVD, the mean (SD) WEMWBS scores of individuals who did not have a long-standing illness (51.14 ± 7.65 vs 47.07 ± 9.54; *p* < 0.05), diabetes (48.44 ± 9.05 vs 46.04 ± 10.25; *p* < 0.05), or high blood pressure (48.63 ± 9.08 vs 47.52 ± 9.47; *p* < 0.05) were significantly higher than those of individuals with such conditions. Meeting PA recommendations was significantly associated with a higher mean WEMWBS score (50.64 ± 7.97 vs 46.06 ± 9.75; *p* < 0.05). Multiple regression analysis of health-related behaviors improved the prediction of mental well-being over and above meeting the recommended PA levels. Mental well-being was strongly correlated with PA adherence in CVD patients. It seems that for patients with CVD, PA should be tailored to meet patients’ health conditions in order to promote mental well-being and improve overall health.

## 1. Introduction

For decades, the physical health of patients with mental illness has been neglected. Studies agree that excess mortality and comorbidity are important in this population [1], with an all-cause mortality rate 4.5 times higher than that of the general population [1]. Thus, a schizophrenic patient has a life expectancy that is 20% lower than that of someone in the general population. According to Saravane [1], the main causes are metabolic syndrome and cardiovascular disease (CVD). CVD and the associated risk factors, as well as mental health problems, represent major challenges for public health. According to World Health Organization (WHO) projections, ischemic heart disease and unipolar depression are the two diseases that will have the greatest burden of disease in developed countries in 2030 [2]. CVD and mental disorders often occur concomitantly [3]. Indeed, studies of patients with ischemic heart disease have found high rates of depression [4,5]. In these patients, the presence of depressive symptomatology is accompanied by a high mortality risk. Hence, it is important to recognize that mental health is an essential component of overall health, particularly in patients with CVD. As we know, mental health is a state of well-being in which individuals can realize their own potential and cope with normal situations in life and the stresses they generate [6]. A person in good mental health can contribute to the community and work productively [6]. Mental health is therefore a state of complete physical, mental, and social well-being and is not simply an absence of mental disorders.

Improving the mental health of individuals represents a social and economic priority. The economic costs of mental disorders/illnesses are very important. A conservative assessment has estimated that the repercussions of mental illness amount to more than $50 billion per year [7]. Rising costs, especially regarding the care of patients with metabolic syndrome and CVD, have caused governments to demand a more efficient management of care in order to reduce the burdens of disability and mortality risk.

The question is how to best promote mental and physical health in patients with CVD. There are a number of measures available to increase the likelihood of more people enjoying good mental health or mental well-being. These interventions—which can be psychological, physical, or even pharmacological—have been used to treat mental disorders associated with metabolic syndrome. Government spending on mental health remains comparatively low. However, given that the pharmacological management of mental disorders has been hampered by low efficacy, high rates of adverse effects, and a substantial economic burden to patients and health systems [8,9], there is a growing need for non-pharmacological therapies to lower the burden of mental disorders.

One such intervention is that of physical activity (PA) [10]. PA has been found to lower the risk of the occurrence of some cancers, diabetes mellitus, and CVD by up to 28% [11], further reducing all-cause mortality by up to 19% [12]. PA has been demonstrated to be an effective therapeutic intervention for depression [13] and metabolic syndrome-related mental disorders [14,15]. According to Ford et. al. [15], physical inactivity is an important potential determinant of the prevalence of metabolic syndrome. Metabolic syndrome greatly increases the risk of type 2 diabetes, CVD, and stroke. Regular PA helps individuals lose weight and maintain a stable weight thereafter. In addition, the adoption of a healthy lifestyle (e.g., avoiding smoking) and diet reduces all of the risk factors associated with metabolic syndrome [16,17,18,19,20,21,22,23]. Vancampfort et. al. [16] have suggested that in patients who smoke, low levels of PA are associated with medium-to-high levels of depression. Low levels of PA have also been associated with risk factors for CVD combined with metabolic syndrome and are a prominent cause of mortality compared with diabetes alone when considering all-cause mortality [24].

The role of PA in mitigating the impact and severity of mental health conditions has also been widely documented [25,26]. Although the relationship between PA and mental well-being is clear, its association with current PA recommendations is poorly studied. In fact, according to a recent report from the US National Center for Health Statistics, only 22.9% of adults aged 18–64 followed the guidelines for PA between 2010 and 2015 [27]. The current globally recommended level of PA for adults aged 18–64 years is at least 150 min of moderate-intensity or 75 min of vigorous-intensity aerobic PA per week, or an equivalent combination of both [28]. These guidelines are designed to provide information and direction for different populations; however, there are no exact data on how adults with metabolic syndrome can adhere to the current PA guidelines. Herring et. al. [29] have reported that a high percentage of CVD patients—including patients with diabetes, cancer, and high blood pressure (BP)—do not achieve the recommended levels of PA. Zachariah et. al. [30] state that this is due to both the effects of exercise intensity and the fitness levels of individuals.

The levels of PA reported to be necessary to lower the risk of mental disorders or improve mental well-being vary, with different studies stating that anywhere between <60 min and 420 min per week is sufficient [31,32,33]. Furthermore, although a dose–response relationship has been shown between PA and other health outcomes [11], it has not been consistently demonstrated with mental well-being [34,35,36]. Ultimately, this has led to mixed results regarding the association between PA recommendations and mental well-being [32,36,37]. The variability in findings may be the result of small sample sizes, a failure to account for baseline mental health, pre-existing major diseases [32,36], or the accuracy of activity reporting and adherence to the WHO recommended guidelines [28].

For this reason, a more precise evaluation of the effects of recommended PA levels on mental well-being would help develop public health recommendations and health promotion campaigns. Although there is increasing interest in the relationship between PA and mental health, including positive mental well-being and happiness [32,33,34,35,36,37,38], this association has not yet been adequately studied in a Scottish population with CVD. Analysis of government-supported national health surveys, such as the UK’s Scottish Health Survey (SHeS), can provide valuable information about the relationship between mental well-being and PA [39]. Thus, the aim of this study was to assess the association between mental well-being and adherence to recommended PA guidelines in a Scottish adult population with CVD.

## 2. Materials and Methods

### 2.1. Study Design and Participants

This analysis is based on data collected as part of the SHeS [40], a nationally representative cross-sectional survey designed to provide data on the health and well-being of Scottish adults aged ≥16 years and children aged 0–15 years, living in private Scottish households [40]. The survey provides comprehensive information on health and health-related risk factors among individuals residing in Scotland that cannot be obtained from other sources every year [40]. Further details of the survey’s methods, questionnaire, and latest report findings can be found on the SHeS website: www.gov.scot/scottishhealthsurvey. For the purpose of this study, we included data on adults aged ≥16 years from SHeS surveys carried out annually between 2014 and 2017 by ScotCen Social Research (Edinburgh), the MRC/CSO Social and Public Health Sciences Unit (Glasgow), the Centre for Population Health Sciences (University of Edinburgh), and the Public Health Nutrition Research Group (University of Aberdeen). We restricted our analysis to only individuals with a CVD diagnosis. Individuals were classified as “any CVD” if they reported a physician’s diagnosis of the following conditions: angina, heart attack, stroke, heart murmur, abnormal heart rhythm, or “other heart condition” [39]. Our observational study followed Strengthening the Reporting of Observational Studies in Epidemiology (STROBE) guidelines.

### 2.2. Sample Characteristics

Personal interviews were carried out by trained interviewers via computer-assisted interviewing to obtain information covering: CVD; long-standing illness such as diabetes, chronic obstructive pulmonary disease (COPD), and high BP; smoking status; number of portions of fruit and vegetables consumed daily; age; and body mass index (BMI) [39]. Fruit and vegetable intake was assessed with the intention of monitoring population-level adherence to the five-a-day recommendation. Smoking status was classified as “current cigarette smoker,” “ex-regular cigarette smoker,” and “never a regular cigarette smoker or never smoked cigarettes at all.” Individuals were classified as having diabetes, COPD, or high BP if they reported a physician’s diagnosis of the same. Area deprivation data were presented in Scottish Index of Multiple Deprivation (SIMD) quintiles [41]. These are based on 31 indicators in six individual domains (current income; employment; housing; health; education, skills, and training; and geographical access to services and telecommunications). We report SIMD in five quintiles; the first and second fifths are the least deprived, and the fourth and fifth are the most deprived.

### 2.3. Mental Health and Well-Being

Well-being was measured using the Warwick–Edinburgh Mental Well-Being Scale (WEMWBS) questionnaire. It comprises 14 items measured on a five-item scale ranging from “1—None of the time” to “5—All of the time,” and is used to assess positive effects (optimism, cheerfulness, and relaxation), satisfying interpersonal relationships, and positive functioning (energy, clear thinking, self-acceptance, personal development, mastery, and autonomy) [42]. When the WEMWBS was developed in the UK, the Cronbach’s alpha score was 0.91 and the test–retest reliability was 0.83 [42]. The lowest score possible is 14 and the highest score possible is 70. A higher WEMWBS score indicates a higher level of mental well-being [42].

### 2.4. PA

PA interview module questions were based on the Allied Dunbar National Fitness Survey [39]. Participants were asked to report any PA lasting ≥10 min and the number of days in the past four weeks on which they had taken part in such activities. The interview questions considered four main types of PA: home-based activities (housework, gardening, building work, and DIY), walking, sports and exercise, and activity at work. The information was collected on the basis of the time spent being active, the intensities of the activities undertaken, and the frequencies at which the activities were performed. The intensity levels of the activities mentioned by the participants were estimated to help assess adherence to the guidelines. A more detailed discussion of how the amount of PA was estimated is provided in the SHeS report [39]. PA was classified as “met” or “unmet” on the basis of moderate or vigorous PA guidelines and the recommended amounts of PA for adults issued by the UK Chief Medical Officer in 2011 [43]. These guidelines, which represent current recommendations, recommend the accumulation of 150 min of moderate activity or 75 min of vigorous activity per week, or an equivalent combination of both, in bouts of ≥10 min.

### 2.5. Ethical Considerations

The SHeS data were analyzed with the permission of the UK Data Service [40]. The survey was approved by the Research Committee for Wales (reference number 12/WA/0261).

### 2.6. Statistical Data Analysis

Analyses were conducted using IBM Statistical Package for Social Sciences (SPSS) software statistics version 25 (IBM Corp, Armonk, New York, USA). We selected age, gender, SIMD quintile, BMI, fruit and vegetable consumption, smoking, PA, long-standing illness [44], high BP [45], diabetes [46], and COPD [47] as confounding variables among the SHeS items that are associated with both mental health and PA. A descriptive analysis was carried out with categorical variables presented as proportions and continuous variables summarized using mean and standard deviation (SD). An independent-samples *t*-test and one-way analysis of variance test were performed to determine any differences in mean WEMWBS scores between categorical variable groups. Cohen’s *d* and partial eta squared (η2) tests were used as measures of effect sizes [48]. Hierarchical multiple regression was used to determine if the addition of the variables of diet, BMI, age, SIMD quintile, gender, smoking status, long-standing illness, diabetes, BP, and COPD improved the prediction of mental well-being over and above meeting PA recommendations alone. In Model 1, we assessed the association of adherence to recommended PA levels on mental well-being without accounting for the additional variables. In Model 2, we assessed whether the addition of the above variables improved the prediction of mental well-being over and above meeting the PA recommendations alone. See Table 4 for full details of each regression model. 

## 3. Results

Of the 23,929 adults aged ≥16 years (10,976 men and 12,953 women) who participated in the SHeS between 2014 and 2017, a total number of 3128 (1547 men and 1581 women) had CVD conditions and represented valid samples that were used in this analysis. About 76% of the study population reported having a long-standing illness. Only 41.8% of the total study population reported meeting the PA guidelines, with males being more physically active compared with females. Never-smokers represented 43.9% of the sample population. The mean age of the sample population was 63.29 years, with the average male age being slightly higher than the average female age. The average BMI of the overall study population was classified as “overweight,” with the average BMI being slightly higher among males. The baseline characteristics of the participants are summarized in Table 1. Fruit and vegetable consumption of female patients was higher than that of males. The prevalence of metabolic syndrome (i.e., diabetes or high BP) was higher in males compared with female patients (in %). The SMID index appeared to be slightly higher among females compared with males. 

An independent-samples *t*-test was performed to determine whether there were any significant differences in WEMWBS scores between categorical variable groups. As seen in Table 2, the WEMWBS scores did not show any significant differences according to gender (*p* > 0.05). However, the other categorical variables—individuals who did not have diabetes, COPD, high BP, or a long-standing illness—were shown to have higher mean (SD) WEMWBS scores.

The differences in mean WEMWBS scores according to the profiles of the participants are summarized in Table 2 and Table 3. Overall, among those with CVD conditions, the mean (SD) WEMWBS scores of individuals who did not have long-standing illnesses were significantly higher than those of individuals who did (51.14 ± 7.65 vs 47.07 ± 9.54; *p* < 0.05). In addition, mean (SD) WEMWBS scores of individuals who did not have diabetes (48.44 ± 9.05 vs 46.04 ± 10.25; *p* < 0.05), COPD (48.58 ± 9.03 vs 43.54 ± 10.21; *p* < 0.05), or high BP (48.63 ± 9.08 vs 47.52 ± 9.47; *p* < 0.05) were significantly higher compared with those of individuals who did not have such conditions. Individuals who achieved the recommended levels of PA had significantly higher mean WEMWBS scores than those who did not (50.64 ± 7.97 vs 46.06 ± 9.75; *p* < 0.05). In addition, the mean (SD) WEMWBS scores of individuals according to the SIMD 2016 quintiles were significantly lower in individuals belonging to the lower quintiles compared with individuals in the higher quintiles. The mean (SD) WEMWBS scores of participants who were non-smokers, ex-smokers, and current smokers were 49.62 ± 8.66, 48.52 ± 8.74, and 43.83 ± 10.23, respectively (Table 3).

Hierarchical multiple regression analysis (*N* = 2200) was performed to determine if the addition of diet, BMI, age, SIMD quintile, gender, smoking status, long-standing illness, diabetes, BP, or COPD improved the prediction of mental well-being over and above meeting PA recommendations alone (Table 4).

The relationship between meeting PA guidelines and WEMWBS scores was explored using hierarchical linear regression accounting for the set of health and sociodemographic characteristics. In Model 1, meeting PA recommendations significantly predicted the WEMWBS score. The addition of health and sociodemographic variables for the prediction of WEMWBS scores (Model 2) led to a statistically significant increase in R^2^ of 0.11, F (10, 2188) = 29.28, *p* < 0.001. Meeting PA guidelines remained a significant predictor of the WEMWBS score after accounting for other factors and covariates in the final model: R^2^ = 0.17, F (11, 2188) = 40.23, *p* < 0.001; adjusted R^2^ = 0.16.

## 4. Discussion

In this study, we found that individuals with CVD who met the current recommendations for PA were more likely to have greater mental well-being. However, according to our findings, only 45.5% of men and 38.2% of women met the recommendations for PA. The WEMWBS scores did not show any significant difference according to gender. Interestingly, after adjustments were made for potential confounders in the hierarchical multiple regression analysis, meeting the current recommendations for PA was an independent predictor of greater mental well-being. Furthermore, we found that this effect was influenced by age, diet, BMI, SIMD quintile, cigarette smoking, COPD, and having a long-standing illness. Recent studies have continued to provide insight into the interplay between PA and improved mental well-being [49,50]. A study by Kim et. al. [34] reported that their study revealed a U-shaped relationship rather than the dose–response relationship between PA and mental well-being observed by other researchers. Also, they found that achieving two to three times the recommended minimum level of PA was an optimal range for a beneficial outcome and that the cutoff value differed according to gender [34]. However, in the current study, there were no differences in terms of adherence according to gender. Such results suggest that further work may be needed to define the real role of gender in terms of PA adherence and the improvement of mental well-being. Our findings add to the growing body of evidence on the role of PA in improving mental well-being, and in particular the importance of meeting or exceeding current daily PA recommendations [51]. However, caution must be taken in generalizing the concept that more PA is better, especially in the context of individuals living with CVD. In this study, the age range of the participants was wide. While the lower exclusion criterion was just 16 years, the average age of the participants was 63 years, and thus PA guidelines must be tailored with this in mind. 

The adverse impact that living with CVD has on mental health has been well documented [4]; however, to date there has been limited research investigating whether the favorable impacts that PA has on mental well-being are seen among individuals living with the many conditions that sit under the umbrella of CVD. In the current study, the prevalence of metabolic syndrome in patients with CVD (i.e., diabetes and high BP) was higher (%) in males compared with female patients, as was BMI and smoking status. In contrast, fruit and vegetable consumption was greater in female patients. In the current study, men were more adherent to PA guidelines than women. Similar results have been reported in previous studies [52]. Uffelen et.al. [52] explained their results by suggesting that motivational factors differ from women to men and therefore recommended that individuals aged >60 years should receive more specified PA guidance to meet their motivation and objectives.

Interestingly, the most important implication of our finding is that even among individuals with CVD, meeting the recommended levels of PA remains an independent predictor of improved mental well-being. This suggests that in addition to improving the outcomes of individuals with CVD [53,54,55], PA is also important in improving their mental well-being. Moreover, given recent findings that associations between mental well-being and CVD may be mediated through biological, behavioral, and psychosocial pathways, individual-level interventions such as achieving the recommended levels of PA may be useful in improving mental well-being and CVD outcomes [56]. An intervention is currently underway to assess the impact of community PA promotion on adults with CVD risk and/or mental health concerns [57]. The effects of PA on overall health are so strong that some authors consider it to be a psychotherapeutic process, particularly in reducing phobias [58], depression, and anxiety [59], and in improving mood [60]. However, epidemiological study data suggest that more than one-half of the population in Western countries is sedentary [61]. The fight against a sedentary lifestyle has become a real public health issue, especially when it comes to special pathological populations. It is well known that PA has a beneficial effect on age-related muscle loss, cognitive performance, and metabolic profile in the healthy population. However, no exact data exist on how PA can improve mental health in those with CVD. Guidelines on CVD prevention in clinical practice [62,63,64] have been described on the basis of different samples and ethnic groups; however, levels of adherence of the whole CVD population to PA and its impact on mental health remain unclear, since the guidelines are directed toward the improvement of overall health and disease prevention or rehabilitation. 

Likely owing to inadequate access to primary prevention and early diagnosis, various socioeconomic determinants have long been associated with CVD and mortality [65,66]. Echoing these landmark studies, the mean WEMWBS score identified for this cohort of individuals with CVD (48.07) was lower than that of the Scottish population average of 49.8 [39]. Within the current study, economic deprivation was found to be inversely associated with mental well-being after adjustments were made for other variables. This finding is consistent with earlier studies that found that economic deprivation has a strong negative correlation with mental well-being [67,68].

Potentially tied to these findings, a higher dietary intake of fruit and vegetables was also found to be a positive predictor of greater mental well-being, agreeing with recent findings that have shown that increased fruit and vegetable consumption is associated with greater mental well-being and a lower risk of depression [69,70]. Similarly, in accordance with prior results [71], we found that smokers, compared with non-smokers and ex-smokers, had lower mental well-being. In addition to CVD, we also identified the notable impact that living with other comorbidities can have on mental well-being.

Our study has some limitations. First, the levels of PA were measured on the basis of self-reports by the participants. Direct biomechanical measurements would have provided a more accurate evaluation of the levels of PA but they are not always feasible in large-scale population studies. Second, as this is a cross-sectional descriptive study, the associations found should be interpreted with caution, and causal inferences should not be made. Third, the assessment made may be prone to recall bias. Fourth, although we primarily analyzed the effect of PA on mental well-being among individuals with physician-diagnosed CVD and adjusted for the confounding of other conditions, we did not adjust for the effects of illness duration, treatment given, or current status of the participants. However, we did consider that such individuals might have mild conditions considering that they were most likely receiving care in hospital.

Overall, there is a clear dose–effect relationship between the level of PA and its health benefits: the more active a person is in terms of frequency, intensity, or duration of activity, the greater the positive effects on health. A recent study found that meeting the recommended aerobic or strength-training PA levels was positively related to 86% higher levels of mental health among young adult male veterans [51]. Exploration of the effects of levels of fitness and the intensity and frequency of PA would help provide new guidelines for the CVD population, both with and without metabolic syndrome.

## 5. Conclusions

Findings from a large nationally representative survey indicate that adherence to the recommended levels of PA is a robust predictor of mental well-being in a population living with CVD. In addition, mental well-being was strongly correlated with PA adherence in CVD patients with metabolic syndrome and a non-healthy lifestyle. It seems that PA for patients with CVD should be tailored to meet individuals’ health conditions in order to promote mental well-being and improve overall health.

## Figures and Tables

**Table 1:** Baseline characteristics.

Characteristics (%)		Male (%)	Female (%)
Whether has longstanding illness	Yes (76.4%)	75.2%	77.5%
	No (23.6%)	24.8%	22.5%
Diabetes	Yes (16%)	18.2%	13.8%
	No (84%)	81.8%	86.2%
COPD	Yes (10.3%)	10.4%	10.2%
	No (89.7%)	89.6%	89.8%
High BP	Yes (51.2%)	53.4%	49.1%
	No (48.8%)	46.6%	50.9%
PA meeting recommendations	Met (41.8%)	45.5%	38.2%
	Unmet (58.2%)	54.5%	61.8%
SIMD 2016 quintiles *	Least deprived (16.4%)	16.5%	16.3%
	4th quintile (20%)	21.8%	18.3%
	3rd quintile (22%)	21.5%	22.5%
	2nd quintile (20.7%)	20.5%	20.9%
	Most deprived (20.9%)	19.7%	22.1%
Cigarette smoking status *	Never smoked (43.9%)	36.8%	50.9%
	Ex-smoker (36%)	41.4%	30.6%
	Current smoker (20.1%)	21.8%	18.5%
		Mean (SD)	
Total portion of fruit and vegetables		2.97 (2.22)	3.17 (2.37)
Age		63.98 (15.59)	62.62 (17.72)
BMI		29.05 (5.29)	28.64 (6.21)

BMI, body mass index; BP, blood pressure; COPD, chronic obstructive pulmonary disease; PA, physical activity; SD, standard deviation; SIMD, Scottish Index of Multiple Deprivation; * *p* ≤ 0.05.

**Table 2:** Mean differences of WEMWBS scores among selected categorical variable groups.

Characteristics (N)		Group Mean (SD) WEMWBS Score	Effect Size Cohen’s d
Whether has longstanding illness (2689)	Yes (2028)	47.07 (9.54)	0.45 *
	No (661)	51.14 (7.65)	
Gender (2689)	Male (1335)	48.30 (9.11)	0.05
	Female (1354)	47.85 (9.44)	
Diabetes (2688)	Yes (405)	46.04 (10.25)	0.26 *
	No (2283)	48.44 (9.05)	
COPD (2688)	Yes (270)	43.54 (10.21)	0.55 *
	No (2418)	48.58 (9.03)	
High BP (2680)	Yes (1336)	47.52 (9.47)	0.12 *
	No (1344)	48.63 (9.08)	
PA meeting recommendations (2674)	Yes (1177)	50.64 (7.97)	0.51 *
	No (1497)	46.06 (9.75)	

N, number of participants; WEMWBS, Warwick–Edinburgh Mental Well-Being Scale; * *p* ≤ 0.05.

**Table 3:** Mean differences of WEMWBS scores among selected categorical variable groups.

Characteristics (N)		Group Mean (SD) WEMWBS Score	95% CI		Effect Size (Eta Squared)
Age in groups * (2689)	16–44 (408)	47.86	46.95	48.77	0.02
	45–64 (857)	46.44	45.74	47.14	
	65–74 (719)	49.08	48.46	49.69	
	75+ (705)	49.16	48.56	49.77	
SIMD 2016 quintiles * (2689)	Least deprived (457)	50.50 (7.81)	49.79	51.22	0.05
	4th quintile (556)	49.59 (8.67)	48.87	50.31	
	3rd quintile (589)	48.88 (9.27)	48.13	49.63	
	2nd quintile (546)	46.78 (8.94)	46.03	47.53	
	Most deprived (541)	44.89 (10.28)	44.02	45.76	
Cigarette smoking status * (2687)	Never smoked (1201)	49.62 (8.66)	49.13	50.11	0.06
	Ex-smoker (948)	48.52 (8.74)	47.96	49.08	
	Current smoker (538)	43.83 (10.23)	42.96	44.70	

CI, Confidence Interval; * *p* ≤ 0.05.

**Table 4:** Full details on each regression model.

Variable	WEMWBS			
	Model 1		Model 2	
	B	Beta	B	Beta
Constant	46.30 *		37.20 *	
PA meeting recommendations	4.42 *	0.24	3.26 *	0.18
Total portion of fruit and vegetables			0.47 *	0.12
Age			0.09 *	0.16
BMI			−0.05	−0.03
SIMD			−0.62 *	−0.09
Whether has longstanding illness			2.86 *	0.14
Gender			−0.20	−0.01
Diabetes			0.50	0.02
COPD			1.80 *	0.06
High BP			0.61	0.03
Cigarette Smoking Status			−1.51 *	−0.13
*R^2^*	0.06		0.17	
*F*	132.67 *		40.23 *	
*∆R^2^*	0.06		0.11	
*∆F*	132.67 *		29.28 *	

B, unstandardized coefficient; Beta, standardized coefficient; * *p* ≤ 0.05.
